# The CYTO-PV: A Large-Scale Trial Testing the Intensity of CYTOreductive Therapy to Prevent Cardiovascular Events in Patients with Polycythemia Vera

**DOI:** 10.1155/2011/794240

**Published:** 2011-05-17

**Authors:** Roberto Marchioli, Guido Finazzi, Giorgina Specchia, Arianna Masciulli, Maria Rosaria Mennitto, Tiziano Barbui

**Affiliations:** ^1^CYTO-PV Coordinating Center, Consorzio Mario Negri Sud, Via Nazionale, 66030 Santa Maria Imbaro, Italy; ^2^Unit of Hematology, Ospedali Riuniti di Bergamo, Bergamo, Italy; ^3^Division of Hematology, University of Bari, Bari, Italy; ^4^Consorzio Mario Negri Sud, Santa Maria Imbaro, Italy

## Abstract

Polycythemia vera (PV) is a chronic myeloproliferative disorder whose major morbidity and mortality are thrombohaemorragic events. Current guidelines advise maintaining hematocrit (HCT) level below 45% in males and 42% in females. Such targets lean on pathophysiological reasoning, while evidence from ECLAP and PVSG-01, the two largest prospective studies in this disease, suggests no difference in the rate of thrombosis in patients maintained at different HCT values below 50%–52%. Cytoreductive therapy in PV (CYTO-PV) is a multicenter, randomized, and controlled trial assess the benefit/risk profile of cytoreductive therapy with phlebotomy or HU aimed at maintaining HCT < 45% versus maintaining HCT in the range 45%–50%. CYTO-PV is being conducted in the framework of the Gruppo Italiano Malattie Ematologiche nell'Adulto (GIMEMA) and is funded by the Italian Drug Agency (AIFA). It is an independent trial with broad recruitment criteria to mimic clinical practice. We describe here the study and its advancement status. *Conclusions*. Clinical research in rare disease can be carried out with limited funds, provided a research hypothesis is felt as clinically relevant by a scientific community willing to share knowledge on the outcome of clinical practice, thus producing scientific results useful to improve treatment and prognosis of patients.

## 1. Background

Polycythemia vera (PV) is defined as a chronic myeloproliferative disorder characterized by clonal proliferation of hematopoietic progenitors resulting in expansion of the erythrocyte mass, and its clinical course is affected by cardiovascular events, the main cause of morbidity and mortality. Arterial thrombotic events are predominant, particularly large vessel arterial events including cerebrovascular accidents, myocardial infarction, and peripheral arterial occlusion [1, 2]. 

Considering the lack of effective therapeutic strategy targeted at the mutated allele JAK2V617F, possibly responsible of the malignancy, current therapies are mainly addressed to prevent the occurrence of thrombosis. 

Based on the complex relationship between thrombosis, hematocrit, and in vitro parameters of tissue perfusion and blood viscosity, the latter has been proved to be an exponential function of the hematocrit. Red cell aggregation increases at high hematocrit (HCT) levels, creating the potential for vascular stasis. As a result, enhanced interplay between platelet, leukocytes and vessel wall increases the risk of thrombosis [3]. The specificity of HCT role has been recently questioned, and the measure of red cell mass has been suggested to evaluate erytrocytosis in PV in order to better assess differences due to contraction or expansion of plasma volume [4]. However, HCT is the most simple measure to be used in clinical practice.

An observational, retrospective study suggested that thrombotic events increase at hematocrit levels higher than 44% [5]. Since this old landmark study, HCT level and its control assumed an essential role in PV. Other small studies have shown increased incidence of vascular occlusive events as well as suboptimal cerebral blood flow in ranges of hematocrit values between 46% and 52% [6]. Accordingly, the use of an aggressive therapeutic HCT target lower than 45% in males and 42% in females has been recommended to be adopted to reduce red blood cells and treat PV patients, preserving them from thrombotic events. Currently, phlebotomy alone or phlebotomy plus cytoreductive drugs in patients at high risk of thrombosis are recommended therapies to control HCT levels [7–11]. So far, no randomized clinical trial has provided evidence-based data assessing the usefulness of tight HCT control in reducing thrombosis. The PVSG-01 and the ECLAP studies, that is, the two largest, prospective observational studies which are available to date, were conducted in 431 PV patients followed for a minimum of 11 years and in 1,638 PV subjects followed for 5 years and found no clear association between HCT values below 52% and risk of thrombosis [12, 13]. 

The American Society of Hematology documented among hematologists a state of uncertainty about how to treat PV: 16% to 29% of hematologists surveyed from either Sweden or the United States used a target hematocrit that was greater than 45% [14]. Similar practice emerged from the ECLAP study showing that only 48% of patients were maintained with hematocrit values below the recommended target values, whereas 39% and 13% of patients had HCT values maintained into the 45%–50% range and greater than 50%, respectively. 

Notwithstanding the lack of trials providing evidence as to the best HCT target to be pursed in PV patients, recommended treatments include maintaining of hematocrit level below 45% in males and 42% in females through phlebotomies and/or cytoreductive drugs [15, 16]. 

CYTO-PV is the first large-scale trial testing the efficacy of different intensity of cytoreductive therapy to prevent thrombotic events in PV patients and has been started in Italy in the year 2008. This paper presents its status of advancement and the characteristics of the first 319 patients enrolled into the trial until September, 15 2010.

## 2. Objective

**Figure 1:**
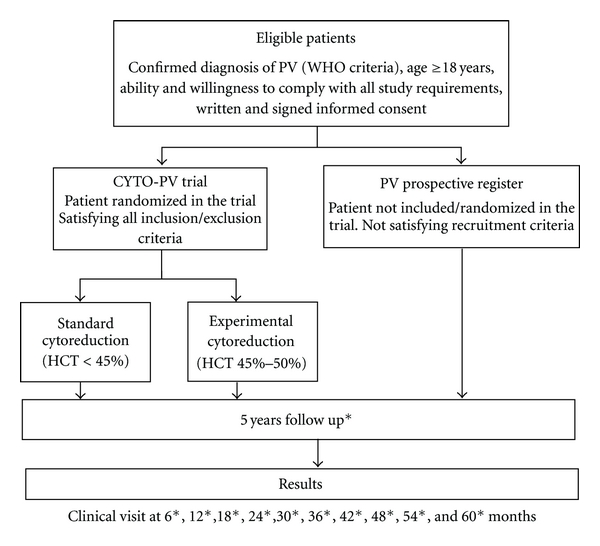
Study design. All patients are to be followed for 5 years with followup visits every 6 months.

The study objective is to assess the benefit/risk profile of cytoreductive therapy, aimed at maintaining hematocrit (HCT) <45% versus maintaining HCT in the range of 45%–50%, in patients with PV. The study design is reported in Figure 1. The population under study will be constituted by women and men aged +18 years with JAK2 positive PV diagnosed according to current clinical practice criteria. Eligible patients will be randomized to maintaining 45% < HCT < 50% versus HCT < 45%.

## 3. Patients/Methods

This is an independent, investigator-promoted trial endorsed by the Gruppo Italiano Malattie Ematologiche nell'Adulto (GIMEMA). The CYTO-PV Steering Committee has the full responsibility for the study design, the collection and analysis of data, and the interpretation and publication of study results. The financial support has been assured by a governmental grant from the Italian Agency of Drugs (AIFA).

### 3.1. Recruitment Criteria

Adults (18+ years) patients with diagnosis of JAK2 positive PV according with WHO criteria and written and signed informed consent are being recruited into the trial. 

Exclusion criteria include (a) known hypersensitivity or contraindication to study treatments, (b) significant liver (AST or ALT > 2.5 times ULN) or renal disease (creatinine > 2 mg/mL), (c) history of active substance or alcohol abuse within the last year, (d) pregnant or lactating women or women not protected from pregnancy by an accepted method of contraception, (e) presence of any life-threatening condition or of any disease that is likely to significantly shorten life expectancy, (f) patient refusing to participate in the study, and (g) any patients' condition that in the opinion of the investigator could lead to poor adherence to the protocol.

The duration of followup is 5 years, and visits will occur every 6 months. Patients will be treated according to the best clinical practice. 

Nonrandomized patients are included into a prospective observational register to assess the generalizability of the CYTO-PV results. Followup visits will be required at the same intervals as the experimental cohort only for recording main patients' characteristics, treatment and the occurrence of major event such as death, thrombotic or hemorrhagic events, and diagnoses of leukemia, solid tumors, or myelofibrosis. All patients will be administered a questionnaire to evaluate the quality of life. 

The trial aims at assessing if long-term cytoreductive treatment with phlebotomy and/or hydroxiurea (HU) aiming at the target of HCT < 45% is more effective than maintaining HCT in the range of 45%–50%. All patients will be treated with the goal to reach and maintain the target HCT established with randomization. It is recommended that patients at high risk of thrombosis (age >65 years or prior thrombosis) or requiring myelosuppressive therapy for progressive thrombocytosis or splenomegaly will be given HU, the myelosuppressive agent of choice. Other treatments, including radiophosphorus, busulphan, pipobroman, and alpha-interferon should be considered as second-choice agents and will be permitted in selected cases according to the best physician's judgment. Phlebotomy should be performed initially by removing 250–500 mL of every other day or twice a week until the target HCT is obtained. HU should be administered initially at a dose of 0.5–1.0 g daily. The patient is followed with weekly blood counts adjusting dose to achieve a platelet count <400,000/mmc. If neutropenia occurs, the dose of HU should be lowered so as not to reduce leukocyte <3,500/mmc. Blood counts at regular intervals (monthly) will establish the frequency of future phlebotomies. Supplemental iron therapy should not be given. 

Because of the open-label design and the type of experimental treatments, no delivery, labeling, or storage of drugs is required. Information on study medication will be collected in the study case report forms (CRF) and kept in the patient's source documentation. Compliance to study treatments will be monitored through the level of HCT maintained during the trial. It is the specific investigator's responsibility to do her/his best to maintain the hematocrit in a range compatible with the randomly allocated target by using pharmacological and/or nonpharmacological “armamentary” currently available. Every effort must be made to ensure that patients remain in the study and are treated for the entire duration of the study in accordance to the HCT target allocated by random procedures. If the medication of a patient is discontinued/changed, the reason(s) for the discontinuation/change are to be collected and recorded in the CRF, and the patients must be followed until study completion with regular followup visits in order to allow a full evaluation of the study endpoints. A permanent discontinuation/change of study randomized targets should be considered only in the best interest of the patients, but the schedule of followup visits will be followed as planned by the study protocol. All treatments proven to be effective for the prevention of cardiovascular disease and treatment of cardiovascular risk factors which are indicated for a patient are positively recommended. Administration of low-dose aspirin is recommended to all patients without contraindications to the drug.

The ethical conduct of the study is regulated by the last revision of the Helsinki Declaration as well as the provisions of the Oviedo Convention. The study protocol is designed to ensure adherence to good clinical practice (GCP) principles and procedures, as described in the following documents and accepted with their signature by investigators:

1. ICH Harmonized Tripartite Guidelines for Good Clinical Practice 1996,
2. Directive 91/507/EEC, the Rules Governing Medicinal Products in the European Community,
3. US 21 Code of Federal Regulations dealing with clinical studies (including parts 50 and 56 concerning informed consent and IRB regulations).

### 3.2. Efficacy and Safety Evaluation

The primary endpoint (PEP) is CV death plus thrombotic events (stroke, acute coronary syndrome (ACS), transient ischemic attack (TIA), pulmonary embolism (PE), abdominal thrombosis, deep vein thrombosis (DVT), and peripheral arterial thrombosis). 

The secondary endpoint is the assessment of the full benefit/risk profile of experimental treatments in terms of efficacy and safety outcome events which include total and CV hospitalizations, incidence of malignancy, PV-related malignancy (progression to myelofibrotic, myelodysplastic, or leukemic transformation), total and nonfatal major hemorrhage (any hemorrhage requiring transfusion, hospitalization, or both), minor bleeding, and any adverse event leading to permanent discontinuation of treatment (e.g., anemia due to phlebotomy).

### 3.3. Study Organization

Data on baseline characteristics, laboratory determination and comorbidity of the patients are collected with the dedicated forms in a web-based, remote data entry system. 

Every 6 months until the end of the study, an electronic followup case report form (e-CRF) will be filled out providing data on clinical events, therapy, and adherence to the study medication. Special forms on death and clinical outcome events of interest have been created to register all of the adverse events occurring during the study. 

The Steering Committee of the CYTO-PV trial has delegated the GCP monitoring aspects of the study to Consorzio Mario Negri Sud. As an investigator-initiated trial with limited research funds, specific, centralized monitoring procedures are activated to assess the reliability of data collection. Data queries are activated through automated checks of the database and visual inspection of CRF. Frequent contacts with the centers will allow to solve all queries timely. The investigators sent monthly through an automatic e-mailing system a report on the quality of their work, the pending issues (e.g., missing CRF, etc.), and the patients they are expected to visit in the next month. Monitoring visits are performed to the research sites after the first 2-3 patients have been enrolled and every 2 years thereafter. Centers with poor compliance to study requirements are monitored with ad hoc site visits.

The architecture of the software developed to manage the clinical trial is based upon the classical 3*-tiers model*, where:

1. the client, with an ordinary web browser (running on a *workstation*) access,
2. the application (located on a *web server*), which interfaces with,
3. a database management system (running on a *database server*) containing the data storage.

A secure web-based software infrastructure based on public-key cryptography by using digital certificates with the secure sockets layer (**SSL**) technology has been created to conduct private, tamper-proof communication with known parties (coordinating center and recruiting sites).

The key features are (a) administration panel for managing centers and accounts, (b) remote input/update and visualization of study case report forms (e-CRFs), (c) generation of real-time reports (tables) on the progress of the study (exportable in doc, xls, and pdf formats), (d) generation of real-time graphs (histograms, lines, and pies) on the progress of the study, (e) notifications via e-mail of actions performed on the website (i.e., user creation, CRF input/update), (f) automatic sending of periodic reports (in PDF format) with the summary of the finalized CRFs and missing or draft CRFs (with no. of days of delay), and (g) log all actions taken online by users (*tracking log*). Each research site as well as the coordinating center maintain paper copies of electronic data forms. Real-time computerized validity and reliability routines have been designed, implemented, and tested. These routines apply the usual data entry validity methods: smart prompting, range checking, data consistency and validity, intelligent field/section skipping, as well as specialized missing data management and automatic summary score calculation routines with manual overrides. The data entry programs employ logical consistency checks based on values from other variables or visit records. Standard operating procedures define the query resolution process, and an audit trail is maintained. SAS is used to generate additional reports regarding completeness and consistency of each database.

### 3.4. Substudies

#### 3.4.1. CY-S1 Substudy

The aim of this substudy is to evaluate in patients with PV the predictive role of *JAK*2^V617F^ allele burden (centrally measured according to real-time quantitative PCR (RTQ-PCR) on clinical outcomes. The primary objective is to ascertain the correlation of the burden of V617F allele, measured at the diagnosis or any time point thereafter in patients with PV with the clinical characteristics at the time of blood sampling. Whenever possible, specific analyses will be carried out to assess such relationship at the time of disease diagnosis and of occurrence of outcome events. The secondary objective is to evaluate time-dependent change in V617F mutational load in patients receiving as cytoreductive therapy only phlebotomy versus HU or other pharmacological therapeutic agents [17, 18].

#### 3.4.2. TG-08 Substudy

This subproject aims to assess the haemostatic potential through the generation of thrombin test, as a predictive test on outcome of bleeding and thrombosis in patients enrolled in CYTO-PV trial. This substudy will include CYTO-PV patients providing written and informed consent. By the use of a fluorogenic thrombin substrate and continuous calibration of each individual sample, it is possible to obtain a thrombin generation (TG) curve (or thrombogram) in plasma, with or without platelets, in an easy routine procedure at high throughput and with an acceptable experimental error (<5%). The parameters of the thrombogram, and notably the area under the curve are useful in assessing bleeding or thrombotic risk and its modification by antithrombotic or haemostatic treatment [19, 20]. Conditions that increase TG cause a thrombotic tendency and conditions that decrease TG prevent thrombosis but, beyond a limit, cause bleeding [21, 22]. The thrombogram can also be used as a tool in the search for new antithrombotics and reflects the haemorrhagic or thrombotic side effects of other drugs. The thrombogram is a promising approach to clinical management of bleeding and thrombotic disease as a tool in drug research and epidemiology. The project requires the collection of blood samples at baseline, every 6 months for 5 years and at the time of occurrence of relevant clinical events (such as thrombosis or major bleeding).

### 3.5. Statistical Aspects

The randomization, managed by the software, is stratified by center, age (>65 years and ≤65 years), and history of thrombosis using the “biased coin” algorithm to establish the probability of allocating patients to the study arms and assure the balance of patients' characteristics within the randomization strata. The randomization scheme and data are kept strictly confidential under the responsibility of the coordinating centre and accessible only to authorized persons. 

The minimum followup duration is set at 5 years. Since in the ECLAP study (median FUP 2.8 years) (9,28), the cumulative incidence of PEP was 5.5% per year in the overall population and 6.95% per year in high-risk PV patients, the expected cumulative rate of the primary endpoint (PEP) in 5 years of followup is expected to be 5% per year in the overall of population (25% in 5 years). The minimum clinically relevant beneficial effect is set at a 30% relative risk reduction of PEP. 1,000 patients are needed to be randomized (alpha = 0.05 (two-tailed), beta = 0.2). Similar to an event-driven trial, the number of expected PEPs which are needed to allow a reliable evaluation of the efficacy of tested interventions is set at 130 in the control group for a study power of approximately 80% at the significance level *α* = 0.05. 

Patients will be followed until the occurrence of a sufficient number of PEPs, unless the trial is stopped early on the basis either of the interim analysis or of new scientific evidences. According to the aforementioned, the study followup will last at least 5 years, provided that:

- the rate of events to be observed during the followup will allow the evaluation of the assumed differences between the treatment arms according to the study design,
- in case the level of significance will not be reached at the interim analysis.

The main analysis will be performed according to an intention-to-treat approach. Baseline characteristics will be presented by treatment groups. Any unbalance for baseline characteristics thought to be of prognostic importance will be considered for multivariate adjustment in the subsequent analyses by fitting to the data a Cox proportional hazards model. The primary analyses of the PEP will be based on the resulting model. Plots of the Kaplan-Meier estimates of the survival curves will be presented. Results will be presented in terms of hazards ratios at their respective 95% confidence interval. The effect of experimental treatments on mortality, bleeding, malignancy, and PV-related malignancy will be assessed in exploratory analysis.

Efficacy in terms of PEP and of all-cause mortality will be monitored using the sequential procedure of Peto. One interim analysis to assess efficacy is scheduled at approximately 1/2 of expected deaths. The corresponding significance level for evidence to stop the trial will be alpha <0.001. Since PV is a rare disease, the significance level of the final analysis will be maintained at *P* < .05. No formal boundaries are proposed for safety, but clear, consistent, and persistent evidence of net harm that overwhelms any benefit will be made apparent to the DSMB. A recommendation by the DSMB to stop the trial will be based on the pattern of treatment effect across all endpoints, as well as the overall benefit/risk ratio of tested treatments.

Primary, secondary, and safety outcome measures will be used for all the subgroup analyses. The effects of the study treatments will be evaluated in the following predefined subgroups of patients: pharmacological versus nonpharmacological cytoreductive therapy, age (above/below median value), HCT (≤40%, 40%–45%, 46%–50%, 51%–55%, >55%), diabetes (y/n), baseline platelet and WBC count (above/below median value), JAK2 status. In the framework of multivariate models allowing for possible unbalance for relevant prognostic covariates, test of interaction will be carried out fitting a statistical model including each of these variables, treatment, and their interaction with treatment. 

The size of the trial will not allow the evaluation of the effects of study treatment on the combined primary endpoint with a power higher than 80% in any of the prespecified subgroups. Therefore, being in the context of exploratory analyses, the evaluation of efficacy will be performed by using two-sided 90% confidence intervals. Tests of interaction will be carried out at the 10% significance level, too. Standard statistical methods will be adopted. ANOVA and t-test will be used for continuous variables. Nonparametric tests will be adopted for continuous variables in case of failure of mathematical transformation for normality. Chi-square and Fisher exact tests will be used for categorical variables as appropriate. Multivariate analysis using either logistic regression or Cox proportional hazards models will be carried out. Time-dependent analysis with time-varying covariates will be carried out as appropriate.

## 4. Results

**Figure 2:**
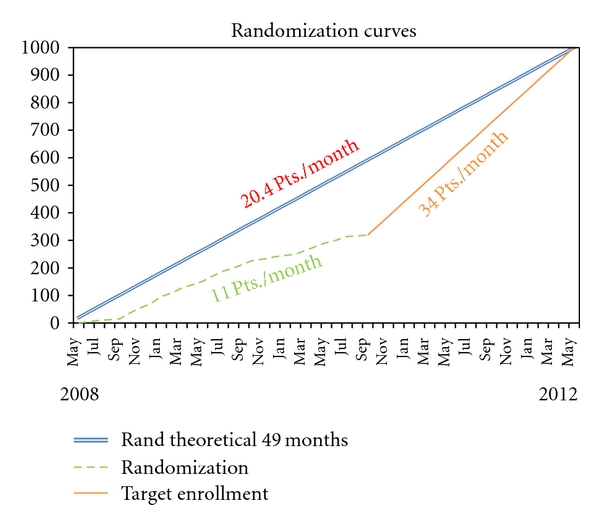
Enrollment trend during the first 2 years. The double line represent the theoretical enrollment trend. The dotted line shows the real enrollment trend, and the straight one represents the future trend to reach the established sample size.

The research network of clinical sites to start the study needed 8 months to collect the adhesion of the 25 centers which are currently active in recruiting patients. Forty-one representatives from 41 divisions of hematology attended the kick off meeting the study. Twenty-five months were needed to obtain the Institute Review Board approval by the 29 hospitals which confirmed their participation to the study and which are to date authorized to recruitment. The research sites are distributed throughout the national territory. The first patient has been randomized on May 26th, 2008. As Figure 2 shows, 319 patients have been enrolled into the trial until September, 15 2010. In the first months of the study, the enrollment rate was low because of the few centers which were authorized to recruit. To date, the recruitment rate has increased, but is still less than optimal. 

Table 1 shows the baseline characteristics of the first 319 patients recruited into the trial at 15 September 2010. Main baseline characteristic are as follows: mean age 65 ± 12 years, females 121 (37.9%). Mean Hct, platelet, red blood, and white blood cell counts were 47.2%, 402 10^6^/mm^3^, 5.7 10^6^/mm^3^, and 10.6 10^3^/mm^3^, respectively. Ninety-nine (31.2%) patients had prior thrombosis (59 arterial, 43 venous, 4 both), while only 16 (5.1%) had a prior bleeding. JAK V617F mutation was present in 307 (96.9%) of patients, only 5 (1.6%) of patients having the JAK2 exon12 mutation. Cytoreductive therapy was performed through phlebotomy as well as prescription of hydroxyurea. In the experimental arm (Hct 45%–50%) 131 (82.4%) patients have been treated with phlebotomy, and 84 (52.8%) with HU. 

**Figure 3:**
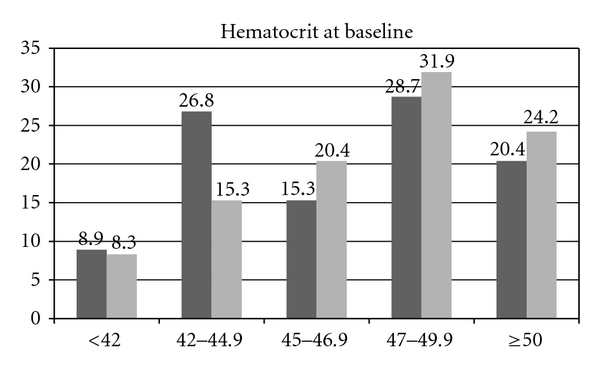
Distribution of HCT values at baseline. The columns indicate HCT levels before randomization for patients allocated in the standard (black) and experimental (grey) arms.

Levels of HCT at baseline are lower than 50% in approximately 78% of patients (Figure 3). 

**Figure 4:**
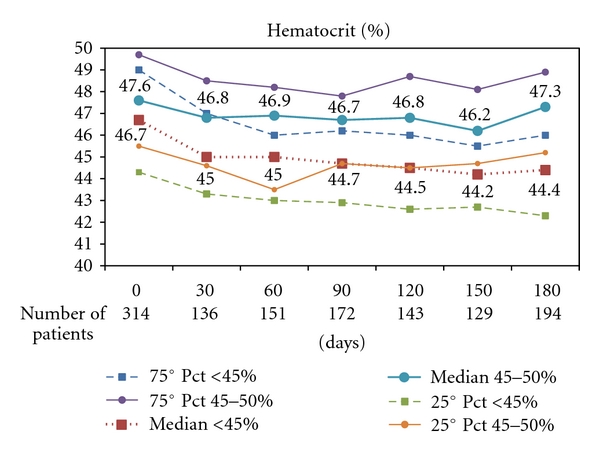
Distribution of Hematocrit (%) values at baseline and during 6 month Followup in each arm of CYTO-PV trial. The heavy dotted red line refers to the group of patients allocated to HCT < 45%; the heavy blue line refers to patients in the experimental arm (HCT 45%–50%).

At 6-month clinical visit in the standard arm (<45%), 130 (82.3%) patients have been treated with phlebotomy and 83 (52.5%) with HU as cytoreductive therapy and therapeutic targets were broadly respected in the two arms for the majority of patients (Table 2), mean HCT values being 44.4 ± 2.3 versus 46.5 ± 2.7. Although the levels of HCT determined by randomization are broadly maintained, there is some difficulty in maintaining HCT levels lower than 44%. Though the HCT levels were significantly different in the two arms, more than 25% of the subjects in each arm were not maintained in the therapeutic range allocated by randomization (Figure 4). At variance with HCT values, the levels of WBC and platelet count were not significantly different between the two treatment arms.

## 5. Conclusions

The current results of CYTO-PV can be summarized under two main headings: (1) the trial is feasible, (2) therapeutic HCT targets allocated with randomization were broadly respected, but some difficulty in maintaining them at the expected level was found.

The results obtained from the assessment of the first 319 patients included in the CYTO-PV trial provide important information about current management of PV in Italy. In fact, 244/314 (77.7%) patients had HCT values under the threshold of 50% before randomization, and 93/314 (29.6%) already had HCT values below 45%. On the other side, HCT levels in the two arms at 6 months after randomization show mean values which are in agreement with the target allocated randomly, but the standard deviation points out some difficulty in maintaining the level of hematocrit strictly in the allocated therapeutic range. 

In a time of evidence-based medicine, uncertainty as to the efficacy of controlling PV by maintaining HCT at specific levels of HCT in terms of clinically relevant outcome events cannot be solved through discussion or pathophysiological reasoning. The CYTO-PV study involving 29 Italian hematological centers represents a simple, but articulated strategy to investigate on this relevant question, assuring a reasonable transfer of the best available validated knowledge: only a multicenter network of clinicians, pathologists, and epidemiologists can provide the “numbers" of subjects and give a sound answer to a clinical need of patients affected by PV; that is, a quasirare disease through the gold standard of randomized clinical trials (RCTs). The experience of the ECLAP has shown that the yield of this strategy is not trivial and is highly cost effective in terms of time and resources. As it should be clear by now, the trial design as well as CYTO-PV comes in as the simplest technical way to deal with uncertainty. Data to be collected, criteria, contents, intensity of followup, and documentation of the events are exactly the same as those which are planned and adopted in routine care. One of the greatest achievements of the multicenter trials with this orientation has been to produce a “core” of data and practices, on which the main analyses will be made, but which at the same time reflect an optimal level of assistance to the majority of patients. The whole PV patient population as it is “naturally” met in (or referred to) specialised centres is considered and suitable for recruitment into a study whose aims are equally important and which complement each other. CYTO-PV registry including patients with established criteria could represent an ideal data-base of the PV population whose prognostic, diagnostic and assistance profiles are compared. Collected data, criteria, contents, intensity of followup, and documentation of the events are exactly the same as those which are planned and adopted in routine care. This leads to producing a “core” of data and practices, on which the main analyses will be made, but which at the same time reflect an optimal level of assistance to the majority of patients. 

Finally, the framework of the collaborative studies (registry and RCT) like CYTO-PV is the ideal setting in which to base more in-depth investigational substudies, with several important advantages:

1. a sufficient number of patients could be recruited more rapidly,
2. it is possible to focus in parallel on various hypotheses, which could complement each other,
3. patients will be “representative” of the population, or at least can be traced back to their original denominators.

The transformation of PV care into a model case for collaborative research is a difficult objective, from the point of view of organisation and availability of resources. As is the case for most clinical conditions which already require for their care an important investment of personnel and facilities, the adoption of a formal research protocol is mainly a “cultural” choice, which has been accepted by the Italian community of haematologists participating to the trial. Let us imagine that the concreteness of these variables is not a hard enough issue so that the trial can be completed and patients are assured the right to be recruited into a care strategy, where certainties and uncertainties are given the specific methodological and concrete attention they deserve [23]. 

##  Steering Committee

T. Barbui (Chairman), G. Finazzi, R. Marchioli, and G. Specchia

##  Trial Management and Secretariat

A. D'Amico, L. Marfisi, R. M. Marfisi, A. Masciulli, M. R. Mennitto, C. Pera, M. Piezzo, A. Polidoro, E. Prete, and M. Scarano.

##  Centers and Investigators

Ancona: professor Pietro Leoni; Bari: professor Giorgina Specchia; Bergamo: professor Tiziano Barbui; Brindisi: Dr. Giovanni Quarta; Cagliari: professor Emanuele Angelucci; Catania: professor Rossella Cacciola; Catanzaro: Dr. Luciano Levato; Cuneo: Dr. Davide Rapezzi; Firenze: professor Alessandro Vannucchi; Foggia: Dr. Silvana Capalbo; Messina: professor Caterina Musolino; Milano (Ospedale Maggiore Policlinico) Dr. Alessandra Iurlo; Milano (Ospedale San Raffaele): Dr. Fabio Ciceri; Monza: professor Enrico Maria Pogliani; Novara: professor Gianluca Gaidano; Orbassano: professor Giuseppe Saglio, Padova: Maria Luigia Randi; Palermo: professor Sergio Siragusa; Pavia: professor Giovanni Barosi; Pesaro: professor Giuseppe Visani; Prato: professor Simone Santini; Reggio Emilia: Dr. Alessia Tieghi; Rionero in Vulture: Dr. Pellegrino Musto, Roma (Università Cattolica Sacro Cuore): professor Valerio De Stefano; Roma (Università La Sapienza): professor Giuliana Alimena, Roma (Regina Elena): Dr. Antonio Spadea; San Giovanni Rotondo: Dr. Nicola Cascavilla; Udine: Dr. Anna Candoni; Vicenza: Dr. Francesco Rodeghiero.

## Figures and Tables

**Table 1:** Baseline characteristics.

Patients with of PV	Total *N* = 319
Age at recruitment	64.6 (11.8)
Sex (Males)	198 (62.1)
Years from diagnosis to enrollment	4.4 (5.5)
0–2	146 (45.8)
3–5	69 (21.6)
6–10	64 (20.1)
>10	40 (12.5)

*Hematology*	
Hb g/dL*	15.2 (1.7)
HCT %*	47.2 (4.6)
Platelets 10^(6)^/mm^3^	402 (184)
RBC 10^(6)^/mm^3^	5.7 (1.1)
WBC 10^(3)^/mm^3^	10.6 (14.9)

*Clinical chemistry*	
Total cholesterol mg/dL	173.1 (41.2)
HDL-cholesterol mg/dL	47.7 (14.2)
LDL-cholesterol mg/dL	99.2 (31.6)
Fasting blood glucose mg/dL	94.1 (22.8)

*Prior cardiovascular events*	
*Prior thrombosis*	99 (31.2)
Arterial	59 (18.6)
AMI	27 (8.6)
Stroke	10 (3.1)
TIA	14 (4.4)
PAT	2 (0.6)
Extremities	5 (1.6)
Other	2 (0.6)
Venous	43 (13.6)
PE	5 (1.6)
DVT	21 (6.6)
SVT	14 (4.4)
Other	11 (3.5)
Arterial and venous	4 (1.3)

*Prior haemorrhagic Events*	16 (5.1)
Major bleeding	6 (1.9)
Brain	1 (0.3)
Stroke	0 (0.0)
GI	4 (1.3)
Minor bleeding	10 (3.1)
Erythromelalgia	17 (5.4)

*Cardiovascular risk factors and comorbidity*	
Hypertension	175 (55.2)
Hypercholesterolemia	55 (17.4)
Diabetes mellitus	29 (9.2)
Current smoker	32 (10.1)
Claudicatio intermittent	2 (0.6)
Coronary arterial disease	29 (9.1)

*Management of PV disease*	
Phlebotomy	261 (82.3)
AntiPLT	266 (83.9)
Aspirin	243 (76.7)
Anticoagulant	41 (12.9)
Hydroxyurea	167 (52.7)
32P	0 (0.0)
Busulphan	2 (0.6)
Chorambucil	0 (0.0)
Pipobroman	14 (4.4)
Interferon	7 (2.2)
JAK2 inhibitors	0 (0.0)
Anagrelide	0 (0.0)
Other cytoreductive drugs	3 (1.0)

*Treatments for CV risk factors*	
Hypocholesterolemic medication	43 (13.6)
Anti diabetic medication	16 (5.1)
Anti hypertensive medication	153 (48.3)

*Cytogenetics *	
Jak2 V617F (positive)	307 (96.9)
Jak2 V617F (%)	50.0 (27.6)
Jak2 V617F (negative)	10 (3.2)
Jak2 Exon12 (positive)	5 (1.6)
Jak2 Exon12 (negative)	18 (5.7)

**Table 2:** Blood cell counts in the two experimental arms of CYTO-PV *at the 6-month after randomization. *

	HCT < 45% *N* = 121	HCT 45–50% *N* = 116	*P* value
HCT %	44.4 (2.3)	46.5 (2.8)	<.0001
White blood cells (10^3^/mm^3^)	9.3 (4.7)	9.3 (4.0)	NS
Platelets (10^3^/mm^3^)	394.4 (169.7)	424.3 (185.0)	NS
